# Pathological Crying and Laughing in Motor Neuron Disease: Pathobiology, Screening, Intervention

**DOI:** 10.3389/fneur.2019.00260

**Published:** 2019-03-21

**Authors:** Eoin Finegan, Rangariroyashe H. Chipika, Stacey Li Hi Shing, Orla Hardiman, Peter Bede

**Affiliations:** Computational Neuroimaging Group, Biomedical Sciences Institute, Trinity College Dublin, Dublin, Ireland

**Keywords:** pathological crying and laughing, pseudobulbar affect, emotional lability, involuntary emotional expression disorder, motor neuron disease, amyotrophic lateral sclerosis, biomarkers, magnetic resonance imaging

## Abstract

Pathological crying and laughing (PCL) has significant quality-of-life implications in amyotrophic lateral sclerosis (ALS); it can provoke restrictive life-style modifications and lead to social isolation. Despite its high prevalence and quality of life implications, it remains surprisingly understudied. Divergent pathophysiological models have been proposed, centered on corticobulbar tract degeneration, prefrontal cortex pathology, sensory deafferentation, and impaired cerebellar gate-control mechanisms. Quantitative MRI techniques and symptom-specific clinical instruments offer unprecedented opportunities to elucidate the anatomical underpinnings of PCL pathogenesis. Emerging neuroimaging studies of ALS support the role of cortico–pontine–cerebellar network dysfunction in context-inappropriate emotional responses. The characterization of PCL-associated pathophysiological processes is indispensable for the development of effective pharmacological therapies.

## Introduction

The terms “pathological crying and laughing,” “pseudobulbar affect,” “emotional lability,” and “involuntary emotional expression disorder” are often used interchangeably depending on geographical location and year of publication. Despite the differences in terminology, recurrent episodes of involuntary or exaggerated emotional expression, particularly in the form of crying and laughing, are common in several neurological conditions including, in order of prevalence: motor neuron disease (MND), traumatic brain injury (TBI), multiple sclerosis (MS), stroke, multiple system atrophy-cerebellar type (MSA-C), Alzheimer's disease (AD), and Parkinson's disease (PD) (1–6).

The disorder is particularly common in motor neuron diseases (MND): amyotrophic lateral sclerosis (ALS) and primary lateral sclerosis (PLS). Prevalence estimates vary, but between one quarter and one half of MND patients are thought to be affected (5, 7–11). While there is a paucity of studies comparing PCL prevalence across the spectrum of MND phenotypes, a recent study confirmed the relationship between clinical upper motor neuron dysfunction and PCL prevalence; PCL was most commonly identified in PLS and UMN-predominant patients (39%), followed by typical ALS (29%) and lower motor neuron (LMN) predominant groups (10%). Consistent with this pattern, there was a single case of PCL in a group of 12 patients with progressive muscular atrophy (PMA) (12). While the manifestations of the episodes may be congruent with the person's contemporary emotional state (10, 13, 14), the magnitude of emotional responses is disproportionate to the emotive stimulus and the social context. Such episodes may cause significant distress, embarrassment, and ultimately may lead to social withdrawal (15).

The primary objective of this review is to systematically appraise PCL-related studies in MND from a dual academic and clinical perspective. We outline established and emerging disease-models based on neuroimaging, neurosurgical and neurophysiological studies. The spectrum of clinical presentations, diagnostic challenges, functional impact, and treatment options are also discussed. We preferentially use the term “pathological crying and laughing” (PCL) in this review, in its broadest sense: encompassing the entire spectrum of presentations. Furthermore, while PCL in ALS is the primary focus of this review, we draw further information from PCL studies in other neurological conditions to discuss unifying, symptom-specific, pathophysiological concepts.

## Methods

A formal literature search was conducted using PubMed/Medline and Embase using the terms “pathological crying and laughing,” “pseudobulbar affect,” “emotional lability,” involuntary emotional expression disorder” separately, and in combination with terms “amyotrophic lateral sclerosis,” “motor neuron disease,” “pseudobulbar palsy,” “clinical trials,” “treatment,” and “pathology.” Only articles in English, published between 1988 and October 2018 were included. A total of 220 articles met these criteria; these were systematically reviewed for information relating to diagnosis, disease-mechanisms, anatomical localization, and treatment options.

### Historical Context and Terminology

The abundance of terms used to describe this syndrome epitomizes the tireless efforts to characterize the underpinnings of both physiological and pathological emotional expression. Charles Darwin observed in 1872 that “certain brain-diseases, as hemiplegia, brain-wasting, and senile decay, have a special tendency to induce weeping” (16). Oppenheim (17) used the term “pseudobulbar affect” (PBA), based on the important observation that the disorder commonly occurs in association with motor features of pseudobulbar palsy, a condition resulting from bilateral corticobulbar tract pathology. Although this association is still well-recognized (13, 18), the term PBA may be misleading; new evidence suggests that corticobulbar tract dysfunction alone is neither necessary, nor sufficient to cause PCL (19–23). “Emotional Lability” (EL) was described by Pierre-Marie as early as 1892, a term still commonly used in the literature (24). The term pathological crying and laughing (PCL) was used by Wilson in his influential essay of 1924, in which he introduced his unifying theory of the disorder (25). More recently, the term “involuntary emotional expression disorder” (IEED) has been proposed to encompass the wider range of emotional symptoms which may accompany the disorder (26).

### Clinical Presentations, Diagnosis and Monitoring

A useful conceptualization of PCL is that symptoms may lie on a spectrum, (27) with infrequent, mood congruent but disproportionate episodes at one end, and frequent, mood incongruent episodes at the other. While there is considerable variability in episode type and character across patients, within individuals episodes tend to be consistent over time in terms of symptom type (i.e., uncontrolled laughing or crying), the context in which the episodes recur, the severity, duration, and the degree of voluntary control that the individual retains over the episodes (26, 28). The wide range of presentations coupled with the lack of unifying terminology, has hampered efforts to establish widely adopted diagnostic criteria. Several criteria have been proposed and later revised, reflecting evolving concepts of the PCL (29). *Poeck's* 1969 criteria (30), focus on episodes that are entirely situation inappropriate or unrelated to the patient's internal emotional state. Recent criteria are less restrictive, encompassing presentations across the entire spectrum (26, 27). Revised criteria proposed by Cummings et al. for “involuntary emotional expression disorder” (IEED) (26) are inclusive of episodes that are either disproportionate to the emotive stimulus or to the individual's internal emotional state. The episodes must represent a distinct change from the patient's emotional reactivity prior to the onset of a neurological disease. These criteria specifically require the exclusion of alternative causes for the symptoms; mood, facial tics, dystonia, or substance effects. More recently, Miller et al. proposed to include supportive features such as the presence of pseudobulbar signs and a proneness to anger (27). The latter reflects accruing observations from several studies (14, 23, 27, 31).

PCL needs to be carefully distinguished from mood disorders through careful clinical evaluation (1, 9, 32). A key difference is that crying in depression or excessive laughter in mania occur in the context of pervasive low or elevated mood, respectively (33). In the case of clinical depression, associated symptoms can also be identified such as anhedonia and insomnia (27, 34). Although some studies report an association between depression and PCL (12), more commonly, no significant association is found (8, 13, 35). The clinical distinction may be particularly challenging in cases where pathological crying co-exists with depressive symptoms (2, 36). Emerging evidence suggests that PCL may respond to selective serotonin reuptake inhibitors (SSRIs) within days, whereas depression typically only responds to pharmacological intervention after several weeks (37).

Similarly to the multitude of diagnostic criteria proposed for PCL, several screening and symptom severity scoring instruments have been developed. While not all of these have been extensively validated, these tools have been used in both academic research and clinical trials. The pathological laughing and crying scale (PLACS) was validated for use in stroke patients with “PBA” in 1993. The authors used it as an efficacy measure in a small placebo-controlled trial of nortriptyline; treatment was associated with symptom reduction (38). The Center for Neurologic Study-Lability Scale (CNS-LS) was introduced as a self-reported measure of “affective lability” in ALS (39). This short, self-administered questionnaire consists of 7 items; 4 relating to labile laughing and 3 relating to crying. The scale evaluates subjective burden of symptoms over the preceding week, in terms of episode burden and severity. While this scale relies on retrospective patient account, it has been shown to be an accurate indicator of episode frequency (40). The CNS-LS has been adopted as an efficacy measure in several recent clinical trials (41–44). Another assessment tool, the “emotional lability questionnaire” (ELQ) has also been validated in ALS. It extends the period over which symptoms are assessed from one up to four weeks prior to screening, which helps to capture patients who experience less frequent episodes (45, 46). In addition to PCL, it also includes a specific section on abnormal smiling. One of the strengths of the ELQ is that it includes the caregiver's perspective, which helps to identify lack of insight if significantly discordant scores are given by the patient and the carer. Interestingly, in ALS, there is significant agreement between patient and caregiver scores, indicating that patients are keenly aware of PCL symptoms (45). This concordance contrasts with behavioral deficits in ALS-FTD, where patient reports may differ substantially from caregivers reports (47).

Although screening instruments are valuable tools for identifying and monitoring PCL, they don't evaluate the impact of PCL on individual patients. There is evidence, that PCL impacts on the quality-of-life and social functioning of affected individuals (48) and may contribute to carer distress (49). There is a growing effort to understand the individualized experiences of patients with PCL (14, 35, 50, 51).

## Disease Models

### Traditional and Revised Hypotheses

The traditional PCL model, proposed by Wilson in 1924 has, until recently been the most widely accepted one (25). Under physiological circumstances, it contends, emotional expression is influenced by both voluntary motor and involuntary emotional centers. These pathways descend onto the medullary “facio-respiratory” centers, which mediate the facial movements and breathing patterns necessary to convey emotion. This model predicts that disruption of the descending voluntary inputs to the brainstem, such as may occur in pseudobulbar palsy, results in disinhibition of involuntary emotional influence on expression. In support of this model, Wilson cited cases of dissociated emotional and voluntary facial expression, such as the observation that some patients with pseudobulbar palsy are unable to make voluntary facial movements but can be observed to smile, laugh or cry in response to emotional stimuli. This phenomenon is usually termed “voluntary facial palsy” (52). The contrary, “mimic palsy” or “emotional facial palsy” is sometimes observed, in which a patient with entirely normal voluntary facial movements, exhibits a complete lack of movement on one side of the face when reacting to emotional stimuli (53).

Parvizi et al. highlighted several limitations of the traditional model (54). Patients with bilateral voluntary facial paralysis, as in Wilson's example do not seem to be excessively prone to PBA, as would be predicted by the “disinhibition” model. Furthermore, patients with severe PBA symptoms are usually able to voluntarily mimic laughing or crying, indicating that involuntary expression can occur alongside intact voluntary control.

The revised model of PCL draws on the increasing appreciation of the role of the cerebellum in cognitive processes (55, 56). This model suggests that the cerebellum plays a key role in gating and modulating emotional output in response to contextual cues from cortical and limbic areas (54). Modulation is believed to be mediated through cortico-ponto-cerebellar pathways. In normal motor control, the cerebellum is known to modulate motor output in response to multiple sensory inputs; disruption of these inputs produces motor modulation deficits, including dysmetria. The involuntary emotional expression resulting from disruption of cortico-ponto-cerebellar emotional circuits has, analogously been termed “affective dysmetria” (57).

### Insights From Imaging and Neurophysiological Studies Across Neurological Diseases

Research over the past 30 years has led to a revision of the traditional model of PCL; studies have provided strong evidence to refute a simple causal relationship between PCL and corticobulbar tract dysfunction (58). Instead, a disruption within a widely dispersed network of emotional control appears to underlie the disorder. Tables 1, 2 provide an overview of studies highlighting key anatomical regions implicated in the pathogenesis of PCL. An MRI-based lesion study in post-stroke pathological crying classified patients based on symptom severity (70). It found a positive pathoanatomical correlation between lesion size and location and pathological crying severity. Bilateral pontine infarcts were associated with greatest severity, while bilateral anterior hemispheric infarcts were associated with moderate PCL severity.

**Table 1:** Neuroanatomical regions implicated in PCL circuitry based on clinical observations.

**References**	**Neurological condition**	**Terminology**	***n***	**Main study findings**	**Anatomical localization**
**CLINICAL CASES**					
Parvizi et al. (3)	Cerebellar cyst	PC	1 PC+	Midline cerebellar cyst	Cerebellum (vermis)
Parvizi et al. (59)	MSA-C	PCL	PCL+: 1 PM and 9 clinical MSA-c	Pathological changes confined to cerebellum, basilar pons and olives	cerebellum and brainstem connections
Chattha et al. (60)	PD with STN-DBS	PBA	1 PBA+	Laughter with DBS stimulation of sub-thalamic nuclei	Basal ganglia: STN
Saini et al. (61)	CPM	PBA	1 PBA+	Basilar pons demyelination	CPC tracts: Pons
Martin et al. (62)	Anti-Yo cerebellar degeneration	PBA	1 PBA+	Breast cancer presenting as uncontrollable crying and motor cerebellar syndrome	Cerebellum
McCullagh et al. (20)	ALS	PCL	10 PCL + 8 PCL- 10 HC	Executive dysfunction in PCL+	Pre-motor frontal cortex
Palmieri et al. (45)	ALS	EL	29 EL+	Correlations: (1) bulbar disease and EL (2) bulbar disease and executive impairment. However, no correlation between PCL and cognitive changes	Extra-motor frontal lobe
Olney et al. (13)	ALS	PCL	21 PCL+, 14 PCL–	Laboratory study: PCL+ had impaired regulation of facial expression	Frontal cortex
Hübers et al. (63)	ALS	PCL	10 PCL+, 10 HC	PCL+ more susceptible to mood-incongruent stimuli than controls, PCL associated with emotional lability/suggestibility	Frontal cortex
**NEUROSURGICAL CASES**					
Krack et al. (64)	PD with STN-DBS	Mirthful laugh	2 PL+	Associated with DBS stimulation of bilateral STN in one patient and right STN in other	Basal ganglia: STN
Okun et al. (65)	PD post-thalamotomy	PBA	1 PBA+	Post-thalamotomy pathological laughing	Basal ganglia
Okun et al. (66)	PD with STN-DBS	PC	1 PC+	Pathological crying with DBS stimulation of left sub-thalamic nucleus	Basal ganglia: STN
Famularo et al. (67)	Cerebellar ependymoma	PL	1 PL+	PC as sole presenting feature of cerebellar vermis tumor abutting the floor of the fourth ventricle	Cerebellum (vermis)
Low et al. (68)	PD with STN-DBS	PC	1 PC+	Pathological crying with DBS stimulation in region of caudal internal capsule (without signs of PBP)	Internal capsule (caudal)
Wolf et al. (69)	PD with STN-DBS	PC	1 PC+	Pathological crying with DBS stimulation of sub-thalamic nuclei	Basal ganglia: STN

*CPC, cortico-ponto-cerebellar pathways; CPM, Central Pontine Myelinolysis; Dx, Diagnosis; HC, Healthy control; MSA-C, Multiple system atrophy, cerebellar type; n, sample size; PBP,Pseudobulbar Palsy; PC, Pathological crying; PL, pathological laughing; PM, Post-mortem/Autopsy; SERT, Serotonin transporter; STN-DBS, Subthalamic nucleus, deep brain stimulation; UMN, Upper motor neuron; WM, White matter*.

**Table 2:** Anatomical conclusions of neuroimaging studies in PCL.

**References**	**Neurological condition**	**Terminology**	***n***	**Main study findings**	**Anatomical localization**
Andersen et al. (70)	Stroke	PC	12 PC+	Isolated, bilateral pontine lesions in most severe cases; bilateral basal ganglia lesions in intermediate group; unilateral subcortical lesions in milder	Pons (raphe nuclei), Basal ganglia, Subcortical WM
Murai et al. (71)	Stroke	PC	6 PC+ 9 PC–	Reduced SERT binding ratios in midbrain/pons in PC	Brainstem (raphe nuclei)
Tateno et al. (72)	TBI	PCL	92 PCL+	PLC associated with traumatic frontal lobe lesions, particularly lateral left frontal lobe	Frontal lobes (esp. left lateral)
Ghaffar et al. (23)	MS	PBA	14 PBA+ 14 PBA–	Greater lesion volume in PBA subjects in brainstem; bilateral inferior parietal and medial inferior frontal; right medial superior frontal	Brainstem; Parietal lobes (bilateral inferior); frontal; basal ganglia
Floeter et al. (10)	ALS	PBA	22 PBA+ 25 PBA– 28 HC	PBA+ (vs. PBA-): reduced FA underlying left motor cortex, Increased MD underlying the frontotemporal cortex, the transverse pontine fibers, and MCP. IC pathology in both groups	widespread disruption of CPC tracts in PBA
Wang et al. (21)	Stroke	PCL	56 PCL+, 56 PCL–	PCL associated with pontine infarcts, particularly paramedian lesions	Pons
Christidi et al. (73)	ALS	PCL	28 PCL+ 28 PCL– 25 HC	PCL+ vs. PCL-: Reduced GM volume: left orbitofrontal cortex, frontal operculum, putamen; and bilateral frontal poles. WM pathology: decreased FA in left cingulum bundle, posterior corona radiata	Frontal cortex: left orbitofrontal; and operculum Cingulate WM

*CPC, cortico-ponto-cerebellar pathways; CPM, Central Pontine Myelinolysis; Dx, Diagnosis; HC, Healthy control; MSA-C, Multiple system atrophy, cerebellar type; n, sample size; PBP, Pseudobulbar Palsy; PC, Pathological crying; PL, pathological laughing; PM, Post-mortem/Autopsy; SERT, Serotonin transporter; IC, Internal Capsule; STN-DBS, Subthalamic nucleus, deep brain stimulation; UMN, Upper motor neuron; WM, White matter*.

Among the likely neurotransmitters involved in the physiological expression of emotion, serotonin and glutamate have received particular attention (27, 71, 74). Evidence for dysfunction within these neurotransmitter pathways in PCL comes from the success of serotonergic (31, 38, 75), and anti-glutamatergic drugs (42) in PCL treatment. The selective serotonin re-uptake inhibitors (SSRIs) and tricyclic antidepressants (TCAs), are thought to work by increasing availability of serotonin at synapses in corticolimbic and cerebellar circuits (32).

A SPECT study found reduced brainstem serotonin receptor (SERT) densities in post-stroke PCL (71), providing further evidence of the role of serotonergic transmission in PCL. Serotonergic neurons from the raphe nuclei have widespread projections from the paramedian brainstem to cortical, subcortical, and cerebellar targets (76, 77). Also consistent with the serotonin hypothesis, lesions involving these nuclei and their projections are frequently associated with PCL (70). An MRI study of PBA in MS patients showed an association between symptoms and lesions in key regions: brainstem, bilateral inferior parietal, and medial frontal regions (23).

There is increasing recognition of the role of sensory deafferentation of the cerebellum in PCL (78). Evidence from neurophysiological studies in PCL suggests that the cerebellum may filter emotional output through a “gate-control” mechanism (79). At a cellular level, cerebellar Golgi cells may play a crucial role in gate-control. It has been demonstrated that Golgi cells, when activated from various peripheral inputs, show decreased firing rate, thereby reducing inhibition of granule cells (80). This finding, suggests, that rather than providing “gain-control,” Golgi cells may act as a “context-specific gate” on transmission through the mossy fiber–granule cell pathway. A study of PCL in MSA-cerebellar type found a prevalence of 36% in this condition, in which clinically-significant cerebellar dysfunction is apparent (59). This prevalence estimate exceeds those of studies in idiopathic Parkinson's disease (5), suggesting that cerebellar pathology is linked to PCL in Parkinsonian disorders. Several neurological and neurosurgical case-reports have linked PCL to cerebellar pathology, especially in association with vermis pathology (3, 62, 67, 81). These studies support the cerebellar gate-control theory of emotional expression, indicating that the disruption to cortico-ponto-cerebellar emotional circuitry may underlie this disorder.

### Insights From PCL Studies in Motor Neuron Disease

Corticobulbar tract dysfunction in ALS has been linked to cognitive impairment, and in particular to executive dysfunction (82). A study of PCL in ALS found an association between PCL and poor performance in executive tasks, implicating pre-frontal cortical areas in the disorder (20). Other studies, in contrast have found no such associations (8, 45). Systematic studies of social cognition in patients with PCL are lacking. Despite the conflicting findings, evidence from imaging and neurophysiology studies support the involvement of frontal cortical dysfunction in PCL (73, 79).

Advanced neuroimaging techniques enable the characterization of symptom-specific structural (83, 84), and functional (85, 86) alterations, providing insights into disease mechanisms (87, 88). Given the high prevalence of PCL in MND, it provides unique opportunities to explore PCL-specific network alterations (58). Floeter et al. used MRI diffusion methods to explore the white matter signature of PCL in ALS and PLS (10). Both ALS and PLS patients exhibited considerable white matter pathology in the corticospinal tracts and the corpus callosum. PCL-associated white matter changes were identified in frontotemporal regions, transverse pontine fibers and the middle cerebellar peduncles. A recent multimodal MRI study by Christidi et al. used the CNS-LS to divide a large group of ALS patients into PCL-positive and PCL-negative groups (73). The PCL-positive group showed significant gray and white matter changes compared with the PCL-negative group. The gray matter assessment found reduced volume of left orbitofrontal cortex, operculum, putamen, and of bilateral frontal poles. White matter analyses revealed diffusion abnormalities in the left cingulum, the posterior corona radiata and in the left middle and bilateral inferior cerebellar peduncles. The finding of cerebellar involvement in PCL in ALS again implicates cerebellar dysfunction in the pathophysiology of PCL, across a range of neurological diseases. While it is challenging to detect cerebellar signs clinically in the presence of pyramidal and lower motor neuron degeneration in ALS, imaging studies suggest that cerebellar degeneration is an important feature of ALS pathology, which is likely to contribute to PCL (89–91).

## Therapeutic Options

### Antidepressant Medication in the Management of PCL

Divergent pharmacological strategies have been explored in the management of PCL. SSRIs and TCAs are the most frequently used off-label medications (75). Surprisingly, we did not identify any placebo-controlled trial of any antidepressants for PCL in ALS. In the absence of robust clinical trial data in ALS, evidence from other neurological conditions, most-commonly stroke, is used to guide treatment. There have been positive results in small placebo-controlled trials of SSRIs including citalopram (92), fluoxetine, (31) sertraline (93) and of the TCA nortriptyline (38), in post-stroke PCL. Case-reports and uncontrolled trials reported symptom improvement with amitriptyline (94) and duloxetine (95) in ALS; memantine in AD (96); and mirtazapine in post-stoke PCL (97). A 2010 Cochrane review of treatments for “emotionalism” after stroke concluded that there is “suggestive but not definitive” evidence that antidepressants reduce frequency of symptoms, although it highlighted “several methodological deficiencies” in available studies (98).

### Management of PCL in Motor Neuron Disease

In 2010, dextromethorphan/quinidine (Dx/Q) became the first FDA-approved treatment for PCL, following more than a decade of research into the potential benefits of the commonly-used anti-tussive for this indication (99). Dextromethorphan acts as a non-competitive glutamate antagonist on NMDA-receptors and as an agonist on sigma receptors (100). When administered alone, it is rapidly metabolized by first-pass metabolism through the cytochrome P450-2D6 system. The addition of the CYP-2D6 inhibitor, quinidine, dramatically increases the bioavailability of dextromethorphan (101). In 2004, a randomized, double-blinded study compared treatment with Dx/Q (30/30 mg twice daily) with dextromethorphan alone in ALS patients with PBA, defined by a CNS-LS score ≥13 (43). The combination not only reduced CNS-LS scores and episode frequency but also led to improvements in quality-of-life measures. Treatment-related side effects including nausea, dizziness, somnolence, and loose stools were relatively common however; about one quarter of patients withdrew from treatment, the majority within 1 week (102). A follow-up study in 2010 assessed whether a lower quinidine dose would reduce adverse effects relative to the earlier trial, while maintaining efficacy (42, 103) The study randomized ALS (*n* = 197) and MS (*n* = 129) patients with PBA to Dx/Q 30/10 mg BID, Dx/Q 20/10 mg BID or placebo BID. Both Dx/Q doses were found to reduce episode frequency, CNS-LS scores and to improve the likelihood of symptom remission compared to placebo. There was a lower discontinuation rate than in the earlier trial. It is interesting to note the considerable placebo response rate across efficacy endpoints in both trials. It must also be pointed-out that CYP450 2D6-poor metabolizers (104) were excluded from the efficacy and safety analyses raising questions about the requirement to screen for this phenotype prior to prescribing (105). Efficacy outcomes were also maintained in a 12-week open label extension study (106). A 52-week open-label study in 553 patients, including 199 patients with ALS, reported no serious drug-related adverse effects (75). However, clinicians must be cognizant of underlying cardiac conditions as quinidine can cause serious QT-interval prolongation (107).

### Limitations of Currently Available Treatments

While the emergence of the first FDA-approved drug for PCL is an important advancement, the effective treatment of PCL remains challenging. Unfortunately, there have been no head-to-head trials of Dx/Q and any commonly used antidepressant. This knowledge-gap is particularly problematic given the current cost of Dx/Q, which may be prohibitive. Not only is the price of the approved combination product higher than alternative options, it is dramatically more expensive than the combined cost of its individual components (108). Finally, although Dx/Q was granted approval by the European Medicines Agency (EMA) in 2013, it was subsequently withdrawn by the manufacturer in 2016, on commercial grounds. (109, 110) A 2017 Cochrane review of “symptomatic treatments” in ALS highlighted emotional lability, as a symptom for which there is a “significant gap” in studies regarding the effectiveness of available treatments (111). There is a pressing and unmet need for robust clinical trials of antidepressants in the management of PCL. Finally, there is evidence that ALS patients and carers, lack awareness of the association between PCL and their underlying neurological condition (112), highlighting the importance of enquiring about PCL symptoms in patients with high-risk conditions, such as ALS.

## Conclusions

Pathological crying and laughing is a shared symptom of many neurological conditions across infective, vascular, inflammatory, and neurodegenerative etiologies. Various terminologies have been used to encompass the heterogeneity of symptoms, which vary in severity, emotional congruity, frequency, and degree of control. After centuries of insightful observations, lesion studies and case reports, neuroimaging methods now provide long-awaited *in-vivo* insights into the specific pathophysiological mechanisms underlying the disorder. Patho-anatomical correlations indicate, that irrespective of the pathology (i.e., neurodegeneration, stroke, demyelination, TBI), the disorder occurs due to disruption in circuits involved in the initiation and modulation of emotional output. Key components of the network include sensori-motor cortical regions and their pontine and cerebellar connections. Further research is needed to elucidate the specific role of individual components within the network and their interactions. Effective symptomatic treatments are available; however, further studies are needed to establish individualized treatment strategies for patients experiencing impaired social or occupational functioning.

## Author Contributions

The manuscript was drafted by EF. RC, SLHS, OH and PB contributed to the conceptualization, editing, and revision of this paper.

### Conflict of Interest Statement

The authors declare that the research was conducted in the absence of any commercial or financial relationships that could be construed as a potential conflict of interest.

## Abbreviations

AD: Alzheimer's disease
ALS: Amyotrophic lateral sclerosis
B.I.D.: Bis in die/twice daily
CNS-LS: Center for Neurologic Study-lability scale
DxQ: Dextromethorphan-quinidine
EL: Emotional lability
ELQ: Emotional lability questionnaire
EMA: European Medicines Agency
FDA: US Food and Drug Administration
LMN: Lower motor neuron
MND: Motor neuron disease
MRI: Magnetic resonance imaging
MS: Multiple sclerosis
MSA-C: Multiple system atrophy-cerebellar type
NMDA: N-methyl-D-aspartate
PBA: Pseudobulbar affect
PC: Pathological crying
PCL: Pathological crying and laughing
PD: Parkinson's disease
PLACS: The pathological laughing and crying scale
PLS: Primary lateral sclerosis
PMA: Progressive muscular atrophy
SPECT: Single-photon emission computed tomography
SSRI: Selective serotonin re-uptake inhibitor
TBI: Traumatic brain injury
TCA: Tricyclic anti-depressant
UMN: Upper motor neuron.

## References

[1] Engelman W Hammond FM Malec JF. Diagnosing pseudobulbar affect in traumatic brain injury. Neuropsychiatr Dis Treat. (2014). 10:1903–10. 10.2147/NDT.S63304 25336956 PMC4200065

[2] Patel N Combs H York M Phan C Jimenez-Shahed J Pseudobulbar affect correlates with mood symptoms in parkinsonian disorders but not amyotrophic lateral sclerosis. J Neuropsychiatry Clin Neurosci. (2018). 30:214–9. 10.1176/appi.neuropsych.17070131 29505320

[3] Parvizi J Schiffer R. Exaggerated crying and tremor with a cerebellar cyst. J Neuropsychiatry Clin Neurosci. (2007). 19:187–90. 10.1176/jnp.2007.19.2.187 17431066

[4] Vidovic V Rovazdi MC Kraml O Kes VB. Pseudobulbar affect in multiple sclerosis patients. Acta Clin Croat. (2015). 54:159–63. 26415311

[5] Brooks BR Crumpacker D Fellus J Kantor D Kaye RE. PRISM: a novel research tool to assess the prevalence of pseudobulbar affect symptoms across neurological conditions. PLoS ONE. (2013). 8:e72232. 10.1371/journal.pone.0072232 23991068 PMC3749118

[6] Phuong L Garg S Duda JE Stern MB Weintraub D. Involuntary emotional expression disorder (IEED) in Parkinson's disease. Parkinson Relat Disord. (2009). 15:511–5. 10.1016/j.parkreldis.2009.01.001 19181560 PMC3524499

[7] Work SS Colamonico JA Bradley WG Kaye RE. Pseudobulbar affect: an under-recognized and under-treated neurological disorder. Adv Ther. (2011). 28:586–601. 10.1007/s12325-011-0031-3 21660634

[8] Lonergan K Burke T Pinto-Grau M Vajda A Heverin M Dockree P Emotional lability in ALS: Delineating the relationship between lability, psychological status, cognition, and behavior. Amyotr Later Scleros Frontotemp Degenerat. (2016). 17:273 10.1080/21678421.2016.1232065/0016

[9] Thakore NJ Pioro EP. Laughter, crying and sadness in ALS. J Neurol Neurosurg Psychiatry. (2017). 88:825–31. 10.1136/jnnp-2017-315622 28572273 PMC5629932

[10] Floeter MK Katipally R Kim MP Schanz O Stephen M Danielian L. Impaired corticopontocerebellar tracts underlie pseudobulbar affect in motor neuron disorders. Neurology. (2014). 83:620–7. 10.1212/WNL.0000000000000693 25008395 PMC4141995

[11] Kuipers-Upmeijer J De Jager AEJ Hew JM Snoek JW Van Weerden TW. Primary lateral sclerosis: clinical, neurophysiological, and magnetic resonance findings. J Neurol Neurosur. (2001). 71:615–20. 10.1136/jnnp.71.5.615 11606672 PMC1737610

[12] Thakore N Pioro E Prevalence, associations and course of depression in ALS: Observations from a large cohort. Amyotrop Lateral Scleros Frontotemp Degenerat. (2014) 15:55–6. 10.3109/21678421.2014.960172/088

[13] Olney NT Goodkind MS Lomen-Hoerth C Whalen PK Williamson CA Holley DE. Behaviour, physiology and experience of pathological laughing and crying in amyotrophic lateral sclerosis. Brain. (2011). 134:3455–66. 10.1093/brain/awr297 22155983 PMC3235565

[14] Ahmed F Murphy J Lomen-Hoerth C Utility of a new pseudobulbar questionnaire (PBAQ) for ALS. Ann Neurol. (2010). 68:S23–S4.

[15] Wynn D Kaye R Hepner A Impact of pseudobulbar affect on health and QoL. Ann Neurol. (2010). 68:S12. 10.1002/ana.22093 20839238

[16] Darwin C Chapter 6: Special Expressions of Man: Suffering and Weeping. The Expression of the Emotions in Man and Animals. New York, NY: D. Appleton and Company (1872).

[17] Oppenheim H Textbook of Nervous Diseases for Physicians and Students by Professor H. Oppenheim of Berlin: English trans-lation by Alexander Bruce. London: T. N. Foulis Publisher (1911).

[18] Tortelli R Cortese R Tursi M D'Errico E Muschitiello C Capozzo R Frequency of pseudobulbar affect (PBA) in an incident ALS cohort: results from a population based registry. Amyotrop Lateral Scler. (2012). 13:128–9. 10.3109/17482968.2012.721231/234

[19] Ironside R. Disorders of laughter due to brain lesions1. Brain. (1956). 79:589–609. 10.1093/brain/79.4.589 13396065

[20] McCullagh S Moore M Gawel M Feinstein A. Pathological laughing and crying in amyotrophic lateral sclerosis: an association with prefrontal cognitive dysfunction. J Neurol Sci. (1999). 169:43–8. 10.1016/S0022-510X(99)00214-2 10540006

[21] Wang G Teng F Chen Y Liu Y Li Y Cai L. Clinical features and related factors of poststroke pathological laughing and crying: a case-control study. J Stroke Cerebrovasc Dis. (2016). 25:556–64. 10.1016/j.jstrokecerebrovasdis.2015.11.003 26683594

[22] Asfora WT DeSalles AA Abe M Kjellberg RN. Is the syndrome of pathological laughing and crying a manifestation of pseudobulbar palsy? J Neurol Neurosurg Psychiatry. (1989). 52:523–5. 10.1136/jnnp.52.4.523 2738597 PMC1032309

[23] Ghaffar O Chamelian L Feinstein A. Neuroanatomy of pseudobulbar affect: a quantitative MRI study in multiple sclerosis. J Neurol. (2008). 255:406–12. 10.1007/s00415-008-0685-1 18297331

[24] Marie P Lec,ons Sur Les Maladies De La Moelle. Paris: G Masson (1892).

[25] Wilson SAK Original papers. Some Probl Neurol. (1924). s1–4:299–333. 10.1136/jnnp.s1-4.16.299 PMC1068199 21611529

[26] Cummings JL Arciniegas DB Brooks BR Herndon RM Lauterbach EC Pioro EP. Defining and diagnosing involuntary emotional expression disorder. CNS Spectr. (2006). 11:1–7. 10.1017/S1092852900026614 16816786

[27] Miller A Pratt H Schiffer RB. Pseudobulbar affect: the spectrum of clinical presentations, etiologies and treatments. Expert Rev Neurotherapeut. (2011). 11:1077–88. 10.1586/ern.11.68 21539437

[28] Gallagher JP. Pathologic laughter and crying in ALS: A search for their origin. Acta Neurol Scand. (1989). 80:114–7. 10.1111/j.1600-0404.1989.tb03851.x 2816272

[29] Sauvé WM. Recognizing and treating pseudobulbar affect. CNS Spectr. (2016). 21:37–43. 10.1017/S1092852916000791 28044945

[30] Poeck K Pathophysiology of emotional disorders associated with brain damage. In: Vinken P Bruyn G editors. Handbook of Clinical Neurology. Amsterdam (1969). p. 3–343.

[31] Choi-Kwon S Han SW Kwon SU Kang DW Choi JM Kim JS. Fluoxetine treatment in poststroke depression, emotional incontinence, and anger proneness: a double-blind, placebo-controlled study. Stroke. (2006). 37:156–61. 10.1161/01.STR.0000190892.93663.e2 16306470

[32] Ahmed A Simmons Z. Pseudobulbar affect: prevalence and management. Ther Clin Risk Manage. (2013). 9:483–9. 10.2147/TCRM.S53906 24348042 PMC3849173

[33] Arciniegas DB Lauterbach EC Anderson KE Chow TW Flashman LA Hurley RA. The differential diagnosis of pseudobulbar affect (PBA). Distinguishing PBA among disorders of mood and affect. Proceedings of a roundtable meeting. CNS Spectr. (2005). 10:1–14; quiz 5–6. 10.1017/S1092852900026602 15962457

[34] Lochead jD Maguire GA Nelson M Pseudobulbar Affect Versus Depression: Issues in Diagnosis and Treatment. Psychiatric Times (2018) p. 35.

[35] Adirim ZL Caga J Ramsey E Zoing M Mioshi E Kiernan MC I can't help that i look sad: The experience of emotional lability in the ALS patient and caregiver. Amyotrop Lateral Scleros Frontotemp Degenerat. (2015). 16:34 10.3109/21678421.2015.1089039/0049

[36] Calvert T Knapp P House A. Psychological associations with emotionalism after stroke. J Neurol Neurosurg Psychiar. (1998). 65:928–9. 10.1136/jnnp.65.6.928 9854975 PMC2170401

[37] Iannaccone S Ferini-Strambi L. Pharmacologic treatment of emotional lability. Clin Neuropharmacol. (1996). 19:532–5. 10.1097/00002826-199619060-00008 8937793

[38] Robinson RG Parikh RM Lipsey JR Starkstein SE Price TR. Pathological laughing and crying following stroke: validation of a measurement scale and a double-blind treatment study. Am J Psychiatry. (1993). 150:286–93. 10.1176/ajp.150.2.286 8422080

[39] Moore SR Gresham LS Bromberg MB Kasarkis EJ Smith RA. A self report measure of affective lability. J Neurol Neurosurg Psychiatry. (1997). 63:89–93. 10.1136/jnnp.63.1.89 9221973 PMC2169647

[40] Smith RA Berg JE Pope LE Thisted RA. Measuring pseudobulbar affect in ALS. Amyotrop Lateral Scleros Other Motor Neuron Disord. (2004). 5:99–102. 10.1080/17434470410020058 15512885

[41] Hammond FM Alexander DN Cutler AJ D'Amico S Doody RS Sauve W PRISM II: an open-label study to assess effectiveness of dextromethorphan/quinidine for pseudobulbar affect in patients with dementia, stroke or traumatic brain injury. BMC Neurol. (2016). 16:89 10.1186/s12883-016-0609-0 27276999 PMC4899919

[42] Pioro EP Brooks BR Cummings J Schiffer R Thisted RA Wynn D. Dextromethorphan plus ultra low-dose quinidine reduces pseudobulbar affect. Ann Neurol. (2010). 68:693–702. 10.1002/ana.22093 20839238

[43] Brooks BR Thisted RA Appel SH Bradley WG Olney RK Berg JE. Treatment of pseudobulbar affect in ALS with dextromethorphan/quinidine: a randomized trial. Neurology. (2004). 63:1364–70. 10.1212/01.WNL.0000142042.50528.2F 15505150

[44] Panitch HS Thisted RA Smith RA Wynn DR Wymer JP Achiron A. Randomized, controlled trial of dextromethorphan/quinidine for pseudobulbar affect in multiple sclerosis. Ann Neurol. (2006). 59:780–7. 10.1002/ana.20828 16634036

[45] Palmieri A Abrahams S Soraru G Mattiuzzi L D'Ascenzo C Pegoraro E. Emotional lability in MND: relationship to cognition and psychopathology and impact on caregivers. J Neurol Sci. (2009). 278:16–20. 10.1016/j.jns.2008.10.025 19103449

[46] Newsom-Davis IC Abrahams S Goldstein LH Leigh PN. The emotional lability questionnaire: a new measure of emotional lability in amyotrophic lateral sclerosis. J Neurol Sci. (1999). 169:22–5. 10.1016/S0022-510X(99)00211-7 10540003

[47] Woolley SC Moore DH Katz JS. Insight in ALS: Awareness of behavioral change in patients with and without FTD. Amyotrop Lateral Scleros. (2010). 11:52–6. 10.3109/17482960903171110 19714539

[48] Colamonico J Formella A Bradley W. Pseudobulbar affect: burden of illness in the USA. Adv Ther. (2012). 29:775–98. 10.1007/s12325-012-0043-7 22941524

[49] Goldstein LH Atkins L Landau S Brown R Leigh PN. Predictors of psychological distress in carers of people with amyotrophic lateral sclerosis: a longitudinal study. Psychol Med. (2006). 36:865–75. 10.1017/S0033291706007124 16490122

[50] Murphy J Duong YN Ahmed F Lomen-Hoerth C A new tool to measure pathological laughing and crying in ALS. Amyotrop Lateral Scleros. (2012). 13:165 10.3109/17482968.2012.721231/303

[51] Young C Aynsley G ALS symptoms, disability and quality of life: literature review and model generation. Amyotrop Lateral Scleros Frontotemp Degenerat. (2014). 15:108–9. 10.3109/21678421.2014.960178/097

[52] Trepel M Weller M Dichgans J Petersen D. Voluntary facial palsy with a pontine lesion. J Neurol Neurosurg Psychiar. (1996). 61:531–3. 10.1136/jnnp.61.5.531 8937357 PMC1074060

[53] Hopf HC Muller-Forell W Hopf NJ. Localization of emotional and volitional facial paresis. Neurology. (1992). 42:1918–23. 10.1212/WNL.42.10.1918 1407573

[54] Parvizi J Anderson SW Martin CO Damasio H Damasio AR. Pathological laughter and crying: a link to the cerebellum. Brain. (2001). 124:1708–19. 10.1093/brain/124.9.1708 11522574

[55] Schmahmann JD Sherman JC. The cerebellar cognitive affective syndrome. Brain. (1998). 121:561–79. 10.1093/brain/121.4.561 9577385

[56] Koziol LF Budding D Andreasen N D'Arrigo S Bulgheroni S Imamizu H. Consensus paper: the cerebellum's role in movement and cognition. Cerebellum. (2014). 13:151–77. 10.1007/s12311-013-0511-x 23996631 PMC4089997

[57] Stoodley CJ Schmahmann JD. Evidence for topographic organization in the cerebellum of motor control versus cognitive and affective processing. Cortex. (2010). 46:831–44. 10.1016/j.cortex.2009.11.008 20152963 PMC2873095

[58] Bede P Finegan E. Revisiting the pathoanatomy of pseudobulbar affect: mechanisms beyond corticobulbar dysfunction. Amyotrop Lateral Scleros Frontotemp Degenerat. (2018). 19:4–6. 10.1080/21678421.2017.1392578 29092641

[59] Parvizi J Joseph J Press DZ Schmahmann JD. Pathological laughter and crying in patients with multiple system atrophy-cerebellar type. Mov Disord. (2007). 22:798–803. 10.1002/mds.21348 17290465

[60] Chattha PK Greene PE Ramdhani RA. Pseudobulbar laughter as a levodopa off phenomenon exacerbated by subthalamic deep brain stimulation. J Clin Movement Disord. (2015). 2:13. 10.1186/s40734-015-0023-6 26788349 PMC4711012

[61] Saini M Mamauag MJ Singh R. Central pontine myelinolysis: a rare presentation secondary to hyperglycaemia. Sing Med J. (2015). 56:e71–3. 10.11622/smedj.2015065 25917480 PMC4415110

[62] Martin AN Dillon PM Jones DE Brenin DR Lapides DA. Anti-Yo mediated paraneoplastic cerebellar degeneration associated with pseudobulbar affect in a patient with breast cancer. Case Rep Oncol Med. (2017). 2017:8120689. 10.1155/2017/8120689 28377827 PMC5362719

[63] Hübers A Kassubek J Gron G Gorges M Aho-Oezhan H Keller J. Pathological laughing and crying in amyotrophic lateral sclerosis is related to frontal cortex function. J Neurol. (2016). 263:1788–95. 10.1007/s00415-016-8201-5 27314961

[64] Krack P Kumar R Ardouin C Dowsey PL McVicker JM Benabid AL. Mirthful laughter induced by subthalamic nucleus stimulation. Movement Disord. (2001). 16:867–75. 10.1002/mds.1174 11746616

[65] Okun MS Heilman KM Vitek JL. Treatment of pseudobulbar laughter after gamma knife thalamotomy. Movement Disord. (2002). 17:622–4. 10.1002/mds.10174 12112225

[66] Okun MS Raju DV Walter BL Juncos JL DeLong MR Heilman K. Pseudobulbar crying induced by stimulation in the region of the subthalamic nucleus. J Neurol Neurosurg Psychiar. (2004). 75:921–3. 10.1136/jnnp.2003.016485 15146017 PMC1739063

[67] Famularo G Corsi FM Minisola G De Simone C Nicotra GC. Cerebellar tumour presenting with pathological laughter and gelastic syncope. Eur J Neurol. (2007). 14:940–3. 10.1111/j.1468-1331.2007.01784.x 17662020

[68] Low HL Sayer FT Honey CR. Pathological crying caused by high-frequency stimulation in the region of the caudal internal capsule. Arch Neurol. (2008). 65:264–6. 10.1001/archneurol.2007.53 18268198

[69] Wolf ME Abdallat M Blahak C Krauss JK. Pathological crying induced by deep brain stimulation of the subthalamic nucleus in Parkinson's disease. J Clin Neurosci.(2017) 45:159–61. 10.1016/j.jocn.2017.08.020 28887071

[70] Andersen G Ingeman-Nielsen M Vestergaard K Riis JO. Pathoanatomic correlation between poststroke pathological crying and damage to brain areas involved in serotonergic neurotransmission. Stroke. (1994). 25:1050–2. 10.1161/01.STR.25.5.1050 7818634

[71] Murai T Barthel H Berrouschot J Sorger D von Cramon DY Muller U. Neuroimaging of serotonin transporters in post-stroke pathological crying. Psychiatry Res. (2003). 123:207–11. 10.1016/S0925-4927(03)00065-9 12928109

[72] Tateno A Jorge RE Robinson RG. Pathological Laughing and Crying Following Traumatic Brain Injury. J Neuropsychiatry Clin Neurosci. (2004). 16:426–34. 10.1176/jnp.16.4.426 15616168

[73] Christidi F Karavasilis E Ferentinos P Xirou S Velonakis G Rentzos M. Investigating the neuroanatomical substrate of pathological laughing and crying in amyotrophic lateral sclerosis with multimodal neuroimaging techniques. Amyotrop Lateral Scleros Frontotemp Degenerat. (2018). 19:12–20. 10.1080/21678421.2017.1386689 29034720

[74] Hornung JP. The human raphe nuclei and the serotonergic system. J Chem Neuroanat. (2003). 26:331–43. 10.1016/j.jchemneu.2003.10.002 14729135

[75] Formella A Work S Colamonico J Kaye R Use of antidepressants and atypical antipsychotics in patients with pseudobulbar affect. J Manag Care Pharm. (2011). 17:271.

[76] Kerr CW Bishop GA. Topographical organization in the origin of serotoninergic projections to different regions of the cat cerebellar cortex. J Comparat Neurol. (1991). 304:502–15. 10.1002/cne.903040313 2022761

[77] Bishop GA Ho RH. The distribution and origin of serotonin immunoreactivity in the rat cerebellum. Brain Res. (1985). 331:195–207. 10.1016/0006-8993(85)91545-8 3986565

[78] Parvizi J Coburn KL Shillcutt SD Coffey CE Lauterbach EC Mendez MF. Neuroanatomy of pathological laughing and crying: a report of the American Neuropsychiatric Association Committee on Research. J Neuropsychiatry Clin Neurosci. (2009). 21:75–87. 10.1176/jnp.2009.21.1.75 19359455

[79] Haiman G Pratt H Miller A. Brain responses to verbal stimuli among multiple sclerosis patients with pseudobulbar affect. J Neurol Sci. (2008). 271:137–47. 10.1016/j.jns.2008.04.017 18504049

[80] Holtzman T Rajapaksa T Mostofi A Edgley SA. Different responses of rat cerebellar Purkinje cells and Golgi cells evoked by widespread convergent sensory inputs. J Physiol. (2006). 574:491–507. 10.1113/jphysiol.2006.108282 16709640 PMC1817778

[81] Pollack IF Polinko P Albright AL Towbin R Fitz C. Mutism and pseudobulbar symptoms after resection of posterior fossa tumors in children: incidence and pathophysiology. Neurosurgery. (1995). 37:885–93. 10.1227/00006123-199511000-00006 8559336

[82] Abrahams S Goldstein LH Al-Chalabi A Pickering A Morris RG Passingham RE. Relation between cognitive dysfunction and pseudobulbar palsy in amyotrophic lateral sclerosis (1997). 62:464–72. 9153602 10.1136/jnnp.62.5.464 PMC486852

[83] Bede P Bokde A Elamin M Byrne S McLaughlin RL Jordan N. Grey matter correlates of clinical variables in amyotrophic lateral sclerosis (ALS): a neuroimaging study of ALS motor phenotype heterogeneity and cortical focality. J Neurol Neurosurg Psychiar. (2013). 84:766–73. 10.1136/jnnp-2012-302674 23085933

[84] Bede P Bokde AL Byrne S Elamin M McLaughlin RL Kenna K. Multiparametric MRI study of ALS stratified for the C9orf72 genotype. Neurology. (2013). 81:361–9. 10.1212/WNL.0b013e31829c5eee 23771489 PMC3772833

[85] Abrahams S Goldstein LH Simmons A Brammer M Williams SCR Giampietro V. Word retrieval in amyotrophic lateral sclerosis: a functional magnetic resonance imaging study. Brain. (2004). 127:1507–17. 10.1093/Brain/Awh170 15163610

[86] Agosta F Canu E Valsasina P Riva N Prelle A Comi G. Divergent brain network connectivity in amyotrophic lateral sclerosis. Neurobiol. Aging. (2013). 34:419–27. 10.1016/j.neurobiolaging.2012.04.015 22608240

[87] Bede P Hardiman O. Lessons of ALS imaging: pitfalls and future directions — a critical review. NeuroImage. (2014) 4:436–43. 10.1016/j.nicl.2014.02.011 24624329 PMC3950559

[88] Bede P Querin G Pradat PF. The changing landscape of motor neuron disease imaging: the transition from descriptive studies to precision clinical tools. Curr Opin Neurol. (2018). 31:431–8. 10.1097/WCO.0000000000000569 29750730

[89] Bede P Elamin M Byrne S McLaughlin RL Kenna K Vajda A. Patterns of cerebral and cerebellar white matter degeneration in ALS. J Neurol Neurosurg Psychiar. (2015). 86:468–70. 10.1136/jnnp-2014-308172 25053771 PMC4392231

[90] Prell T Grosskreutz J. The involvement of the cerebellum in amyotrophic lateral sclerosis. Amyotrop Lateral Scleros Frontotemp Degenerat. (2013). 14:507–15. 10.3109/21678421.2013.812661 23889583

[91] Al-Sarraj S King A Troakes C Smith B Maekawa S Bodi I. p62 positive, TDP-43 negative, neuronal cytoplasmic and intranuclear inclusions in the cerebellum and hippocampus define the pathology of C9orf72-linked FTLD and MND/ALS. Acta Neuropathol. (2011). 122:691–702. 10.1007/s00401-011-0911-2 22101323

[92] Andersen G Vestergaard K Riis JO. Citalopram for post-stroke pathological crying. Lancet. (1993). 342:837–9. 10.1016/0140-6736(93)92696-Q 8104273

[93] Burns A Russell E Stratton-Powell H Tyrell P O'Neill P Baldwin R. Sertraline in stroke-associated lability of mood. Int J Geriatr Psychiatry. (1999). 14:681–5. 10489659

[94] Szczudlik A Slowik A Tomik B. [The effect of amitriptyline on the pathological crying and other pseudobulbar signs]. Neurol Neurochirurg Polska. (1995). 29:663–74. 8584093

[95] Ferentinos P Paparrigopoulos T Rentzos M Zouvelou V Evdokimidis I Duloxetine for pathological laughing and crying in amyotrophic lateral sclerosis. Eur Neuropsychopharmacol. (2009). 19:S409 10.1016/S0924-977X(09)70632-7 19785915

[96] Prokšelj T Jerin A Kogoj A. Memantine may affect pseudobulbar affect in patients with Alzheimer's disease. Acta Neuropsychiatr. (2013). 25:361–6. 10.1017/neu.2013.14 25287877

[97] Kim SW Shin IS Kim JM Lim SY Yang SJ Yoon JS. Mirtazapine treatment for pathological laughing and crying after stroke.Clin Neuropharmacol. (2005). 28:249–51. 10.1097/01.wnf.0000185825.34819.ba 16239769

[98] Hackett ML YM Anderson CS Horrocks JA House A Pharmaceutical interventions for emotionalism after stroke 2010. Cochrane Database Syst Rev. (2010) 17:CD003690 10.1002/14651858.CD003690.pub3 20166068

[99] Smith RA. Dectromethorphan/quinidine: a novel dextromethorphan product for the treatment of emotional lability. Expert Opin Pharmacother. (2006). 7:2581–98. 10.1517/14656566.7.18.2581 17150011

[100] Rosen H. Dextromethorphan/quinidine sulfate for pseudobulbar affect. Drugs Today. (2008). 44:661–8. 10.1358/dot.2008.44.9.1258664 19137121 PMC2872986

[101] Zhang Y Britto MR Valderhaug KL Wedlund PJ Smith RA. Dextromethorphan: enhancing its systemic availability by way of low-dose quinidine-mediated inhibition of cytochrome P4502D6. Clin Pharmacol Ther. (1992). 51:647–55. 10.1038/clpt.1992.77 1611804

[102] Smith RA Brooks BR. Treatment of pseudobulbar affect in ALS. Lancet Neurol. (2005). 4:270. 10.1016/S1474-4422(05)70058-2 15847840

[103] Brooks B Cummings J Pioro E Schiffer R Wynn D Hepner A Pharmacokinetic/pharmacodynamic modeling of dextromethorphan/quinidine for a study in pseudobulbar affect. Ann Neurol. (2008). 64:S38–S. 10.1002/ana.21502

[104] Gaedigk A Sangkuhl K Whirl-Carrillo M Klein T Leeder JS. Prediction of CYP2D6 phenotype from genotype across world populations. Genet Med. (2016). 19:69. 10.1038/gim.2016.80 27388693 PMC5292679

[105] Katz J AVP-39: a successful randomized clinical trial on a supportive therapy for ALS. Neurology. (2004). 63:1345 10.1212/01.WNL.0000145385.55983.1E

[106] Pioro E Brooks B Cummings J Schiffer R Wynn D Hepner A Persistent efficacy of dextromethorphan (DM)/Quinidine (Q) for pseudobulbar affect (PBA): Results from a 12-Week, open-label extension (OLE) study. Neurology. (2010). 75:380 10.1212/WNL.0b013e3181e6e52d

[107] Schoedel KA Morrow SA Sellers EM. Evaluating the safety and efficacy of dextromethorphan/quinidine in the treatment of pseudobulbar affect. Neuropsychiatr Dis Treatment. (2014). 10:1161–74. 10.2147/NDT.S30713 25061302 PMC4079824

[108] Cruz MP Nuedexta for the treatment of pseudobulbar affect: a condition of involuntary crying or laughing. PT. (2013). 38:325–8. PMC3737988 23946627

[109] European Medicines Agency The Marketing Authorisation for Nuedexta has Been Withdrawn at the Request of the Marketing-Authorisation Holder 2016. [30/10/2018]. Available online at: https://www.ema.europa.eu/en/medicines/human/EPAR/nuedexta#overview-section.

[110] Motor Neurone Disease Association. Neudexta; Withdrawal of Marketing Authorisation Mndassociation.org Available online at: https://www.mndassociation.org/research/mnd-research-and-you/research-into-treatments/nuedexta/ (Accessed October 15, 2018).

[111] Ng L KF Young CA Galea M Symptomatic treatments for amyotrophic lateral sclerosis/motor neuron disease 2017. Cochrane Database Syst Rev. (2017) 1:CD011776 10.1002/14651858.CD011776.pub2 28072907 PMC6469543

[112] Wicks P Frost J. ALS patients request more information about cognitive symptoms. Eur J Neurol. (2008). 15:497–500. 10.1111/j.1468-1331.2008.02107.x 18325023

